# Predicting VO_2_max in Children and Adolescents Aged between 6 and 17 Using Physiological Characteristics and Participation in Sport Activities: A Cross-Sectional Study Comparing Different Regression Models Stratified by Gender

**DOI:** 10.3390/children9121935

**Published:** 2022-12-09

**Authors:** Vilelmine Carayanni, Gregory C. Bogdanis, Elpis Vlachopapadopoulou, Dimitra Koutsouki, Yannis Manios, Feneli Karachaliou, Theodora Psaltopoulou, Stefanos Michalacos

**Affiliations:** 1 School of Administration Economics and Social Sciences, Department of Tourism Administration, University of West Attica, 28 Saint Spyridonos Str., 12243 Egaleo, Greece; 2 School of Physical Education & Sports Science, National and Kapodistrian University of Athens, 41 Ethnikis Antistaseos Str., Daphne, 17237 Athens, Greece; 3 Department of Endocrinology-Growth and Development, Children’s Hospital P. & A. Kyriakou, Thivon & Levadeias Str., Ampelokipoi T.K., 11527 Athens, Greece; 4 Department of Nutrition & Dietetics, School of Health Science & Education, Harokopio University, 70 El Venizelou Ave. Kallithea, 17671 Athens, Greece; 5 Department of Hygiene, Epidemiology and Medical Statistics, School of Medicine, National and Kapodistrian University of Athens, 75 Mikras Asias Str., 11527 Goudi, Greece

**Keywords:** children, adolescents, VO_2_max, multiple linear regression, machine learning, body mass Index (BMI) category, gender, organized sports activity

## Abstract

Background: The aim of this study is to use different regression models to capture the association between cardiorespiratory fitness VO_2_max (measured in mL/kg/min) and somatometric characteristics and sports activities and making better predictions. Methods: multiple linear regression (MLR), quantile regression (QR), ridge regression (RR), support vector regression (SVR) with three different kernels, artificial neural networks (ANNs), and boosted regression trees (RTs) were compared to explain and predict VO_2_max and to choose the best performance model. The sample consisted of 4908 children (2314 males and 2594 females) aged between 6 and 17. Cardiorespiratory fitness was assessed by the 20 m maximal multistage shuttle run test and maximal oxygen uptake (VO_2_max) was calculated. Welch *t*-tests, Mann–Whitney-U tests, X^2^ tests, and ANOVA tests were performed. The performance measures were root mean square error (RMSE), mean absolute error (MAE), and coefficient of determination (R^2^). All analyses were stratified by gender. Results: A comparison of the statistical indices for both the predicted and actual data indicated that in boys, the MLR model outperformed all other models in all indices, followed by the linear SVR model. In girls, the MLR model performed better than the other models in R^2^ but was outperformed by SVR-RBF in terms of RMSE and MAE. The overweight and obesity categories in both sexes (*p* < 0.001) and maternal prepregnancy obesity in girls had a significant negative effect on VO_2_max. Age, weekly football training, track and field, basketball, and swimming had different positive effects based on gender. Conclusion: The MLR model showed remarkable performance against all other models and was competitive with the SVR models. In addition, this study’s data showed that changes in cardiorespiratory fitness were dependent, to a different extent based on gender, on BMI category, weight, height, age, and participation in some organized sports activities. Predictors that are not considered modifiable, such as gender, can be used to guide targeted interventions and policies.

## 1. Introduction

Cardiorespiratory fitness is an important component of physical fitness and reflects the overall capacity of the cardiovascular and respiratory system to perform aerobic work. Measurement of maximal oxygen uptake (VO_2_max) provides a robust index of cardiorespiratory fitness in individuals of all ages. Recent studies suggest that VO_2_max may be used as a powerful health marker, while high levels of cardiorespiratory fitness may have a protective effect on the cardiovascular system from an early age [1,2,3,4]. For the purposes of strategies prevention, it is very important to define the modifiable factors that contribute to decreases in VO_2_max.

A common method of assessment of cardiorespiratory fitness in children and adolescents is the performance of the 20 m multistage shuttle run test [5]. This is an easy-to-perform test with which a large number of individuals (~10 at a time) can be tested simultaneously. Using this test, it has been shown that obese children in several countries have low VO_2_max [2], but there is a sparsity of data on cardiorespiratory fitness in relation to obesity [6,7,8,9,10,11,12]. In addition, studies indicate that even maternal prepregnancy BMI is negatively correlated with cardiorespiratory fitness [13].

It has been found that in children, the development of VO_2_max depends on age and is influenced by growth during puberty [14,15]. Hormonal changes tend to affect various parameters of physical fitness, including VO_2_max. Previous studies have indicated that the advancement of chronological age [14] and nearing the age of menarche are negatively correlated with VO_2_max values [14,15], although contradictory results have been presented, depending on the age group studied [16]. Many studies have indicated that physical activity is relatively well-correlated with aerobic capacity [17,18,19,20]. Moreover, only a few studies have taken into account the relationship between different sports activities and VO_2_max [21,22,23,24,25].

Several studies have indicated that gender is an important covariate on VO_2_max levels. There are several sex-specific factors that influence VO_2_max, such as the timing of puberty, body composition, and growth patterns. Previous observational studies in children and adolescents have reported higher VO_2_max in boys than in girls [26,27,28]. In addition, many studies have indicated that height and weight are significant predictors of VO_2_max [29,30,31,32,33]. To our knowledge, there is limited information about the differences between boys and girls, or between children and adolescents, in terms of BMI categories, sports activities, ages, and parental BMI.

Statistical modeling in the field comprises different machine learning methods with different predictor variables, the choice of which seems to have a large influence on the model’s predictive performance [34]. Therefore, classic regression models and other supervised machine learning algorithms were used to predict VO_2_max. Traditional regression models, such as multiple linear regression (MLR) models, have several advantages that allow researchers to account for all of these potentially important covariates in a single model, which may lead to a more accurate and precise understanding of the association of each covariate with the dependent variable. Nevertheless, problems may result in inaccurate predictions and conclusions. Because linear regression assumes a linear relationship between dependent and independent variables, it may fail to properly fit complex datasets [35]. Quantile regression modeling is an extension of linear regression modeling, which is used when the conditions of linear regression are not met; however, these conventional methods are prone to overfitting, which occurs when the established model is unable to generalize from the data used to build the model.

Machine learning (ML) methods can be categorized into “white box” regression-based methods and “black box” regression-based methods. The first category comprises the different penalized regression methods, such as ridge regression or regression trees, which produce more interpretable models. In addition, black-box methods lead to less-interpretable models (e.g., support vector machine models or neural network models). Although the methods in these two categories vary considerably, all machine learning algorithms, in general, prioritize predictive accuracy, which entails some similarities in characteristics that set them apart from traditional statistical methods [36].

In this study, predictability, as well as transparency and the direct interpretability of the relationship between BMI category and VO_2_max, while controlling for the above-mentioned variables in the statistical modeling, have been prioritized. These priorities led us to compare traditional statistical models with the above-mentioned machine learning techniques to capture the association between the above-mentioned variables and to predict VO_2_max levels. To our knowledge, there are no other studies in the field comparing traditional methods, such as MLR and QR, with “white box” regression-based methods, such as RR, RT, and “black box” regression-based methods, such as the MLP method and the kernels-based method (SVR), stratified by sex. Thus, the aim of this study was to build different traditional and ML models by utilizing a combination of physiological characteristics and sports-activities-participation characteristics to predict VO_2_max by sex in children and adolescents and to identify the relevant and irrelevant predictors. By eliminating the redundant variables and comparing the most relevant predictors by sex, we were able to increase prediction accuracy.

This paper is divided into four sections. Section 2 describes the models used, the study design, and the statistical analysis procedure. Section 3 provides the results, followed by discussion in Section 4.

## 2. Methodology

### 2.1. Models’ Descriptions

#### 2.1.1. Multiple Linear Regression (MLR)

Linear regression is a common and well-understood method for modeling and predicting the mean of a continuous response variable, conditional on a set of predictor variables [37]:

Y = a + b_1_X_1_ + b_2_X_2_ + … + b_n_X_n_ (1)

Here, Y is the dependent variable, and X_1_,…,X_n_ are the n independent variables. Several studies in the field have used MLR to model and predict VO_2_max [29,30,31,32].

#### 2.1.2. Quantile Regression (QR)

Quantile regression is a procedure used to model the cut points of the cumulated conditional probability distribution of a dependent variable as a function of some covariates. In quantile regression models, the quantiles of a response variable are assumed to be linearly associated with a set of conditioning variables [38]. In general, this translates into a non-linear relationship between the dependent and the independent variables, considering the whole distribution, expressed as follows:

${\mathrm{Qy}}_{\left.i\right|}x_{i}\left(\theta \right)$, where θ is the θ-th conditional quantile of Y_i_ given X_i_.

When covariates are present, estimating the conditional quantile for the ith observation is a question of fitting a parameter vector, so that the expected loss function is minimal:

(2) $${\mathrm{Qy}}_{\left.i\right|}x_{i}\left(\theta \right)=X_{i}^{\prime }\beta \left(\theta \right)$$

Quantile regression is used by researchers for modeling and predicting VO_2_max [39,40] because it does not assume underlying normality but is more robust in regard to normal errors and outliers.

#### 2.1.3. Ridge Regression (RR)

Ridge regression is a type of regularized regression. By applying a shrinkage penalty, the coefficients of many variables may be reduced to almost zero, while still being retained in the model. Ridge regression (RR) was proposed to analyze multivariate data that suffer from multicollinearity, where the variance and covariance of the regression coefficients increase in the X ′X matrix [41,42,43,44]. To eliminate this problem, variance and covariance can be decreased by adding a ridge trace (lambda) parameter to the diagonal elements of the X ′X matrix. The ridge trace value should be 0 ≤ λ ≤ 1 [41,42,43]. If the λ is zero, the parameter estimation is the same as OLS. The parameter estimation of RR in matrix notation can be stated as $\hat{\beta }$ = (X ′X + λI) ^−1^X ′Y. Ridge regression has also been used by some authors to predict VO_2_max because of potential problems of multicolinearity in some predictors [32].

#### 2.1.4. Support Vector Regression (SVR)

Support vector regression (SVR) is widely used in regression problems with high dimensions. It is a kernel-based method, which can translate nonlinear regression to linear regression problems. Due to the strong generalization capability of Gaussian kernels, the SVR model constructed by Gaussian kernel functions has been widely applied in the forecasting field [45].

In contrast to the ordinary least squares method, the objective function of the SVR method aims to minimize the coefficients, w_i_ by using a different loss function, called the ε-insensitive loss function, we can perform regression, as follows:

(3) $$\mathrm{Minimize}:\;t(w,\xi )=½\;\|w^{2}\|+\sum_{i=1}^{m}(\xi_{i}+\xi_{\iota }^{*})\;\left\{(\Phi \left(x_{i}),w\right)+b\right\}-{\;y}_{i}\;\leq \;\varepsilon \;–{\;\xi }_{i}\;y_{i}-\left\{\left(\Phi \left(\mathrm{xi}\right),\;w\right)+\;b\right\}-\;\mathrm{yi}\;\leq \;\varepsilon \;–\xi_{i}^{*}\;\_\xi_{i}\geq 0\;(i\;=\;1,\ldots ,m),$$

where m is the number of training patterns, y_i_ = ±1,

and C is the penalty value

The error term is handled in the constraints, where we set the absolute error less than or equal to a specified margin, called the maximum error, ϵ (epsilon). Epsilon can be tuned to achieve the desired accuracy of our model. In addition, the ϵ-sensitive error function is not affected by small errors and is less affected by large errors and, therefore, it is more robust to outliers [46]. A kernel is a function returning the inner product (Φ(x), Φ(x′) between the images of two data points x, x′ in the feature space, where Φ is the mapping of the input data into a high-dimensional feature space defined by a kernel function [46].

In this paper, we used the linear kernel, the radial basis function kernel, and the polynomial kernel.

- The linear kernel implements the simplest of all kernel functions:

k(x, x′) = (x, x′)(4)

- The radial basis function implements

(5) $$k(x,\;x\prime )=\exp(-\frac{\left\|x-x\prime \right\|}{{2\sigma }^{2}})$$

- The polynomial kernel implements

(6) $$k(x,\;x\prime )=(x,\;x\prime )=({\gamma (\mathrm{xx}\prime )+b)}^{d}$$

where *d* is the degree of the polynomial term, and γ is the structural parameter in the polynomial and RBF kernels. Different d, γ, and penalty coefficient C values are tested in the paper. A number of studies have used SVR regression to estimate VO_2_max in combination with other methods [47,48,49,50].

#### 2.1.5. Artificial Neural Networks for Regression (ANN)

An artificial neural network is defined as a network that is composed of a large number of simple processors (neurons) that are massively interconnected, operate in parallel, and learn from experience (examples). These are the primary known characteristics of neural systems in biology and medicine that are easiest to exploit in artificial neural systems. The purpose of using artificial neural networks for regression instead of multiple linear regression is that the multiple linear regression can only learn the linear relationship between the features and the target, whereas artificial neural networks that do not have a priori assumptions about the relations between independent variables and the target can learn the complex non-linear relationship [51,52]. Multi-layer perceptron (MLP) is a supplement of a feed-forward neural network that consists of three types of layers: the input layer, the output layer, and the hidden layer. The input layer receives the input signal to be processed.

#### 2.1.6. Regression Trees (RT)

Other branches of the categories of regression-based ML methods that produce easy-to-interpret models are tree-based methods, such as regression trees. The most widely known methodology for building decision trees is the classification and regression tree (CART) algorithm proposed by Breiman et al. [53]. The main objective of CART is to model a response variable from a set of decision rules imposed on the independent variables. CART uses binary recursive partitioning, so that these decision rules are determined by repeatedly partitioning the data into successively smaller groups (i.e., subgroups) with binary splits based on one single predictor variable [54].

For this method, the best split for each exogenous variable is determined by the one that yields maximum homogeneity (i.e., the lowest residual sum of squares) at each partition. In regression problems, CART predicts outcomes based on the mean of the response values for all observed data that fall in that subgroup regression [54].

### 2.2. Data Collection

This was a cross-sectional study and the study population comprised schoolchildren attending primary and secondary schools located in several municipalities across Greece. A stratified cluster-sampling method, based on the total number of primary school and high school students in Greece, was employed. The study was conducted according to the guidelines of the 1975 Declaration of Helsinki, as revised in 1996, and approved by the Greek Ministry of National Education, the Greek Ministry of Health, and the Hellenic Data Protection Authority (ethical code MIS 301205). Seventy-six primary schools and sixty-four secondary schools were included. Details for all regions and prefectures included in the sample and respective participation rates are provided in Supplementary Materials Table S1.

The children and their parents were informed in writing about the aim of the study and the possible risks involved, and the parents signed an informed consent form. Children and adolescents with severe chronic illnesses, i.e., malignancies, diabetes mellitus, rheumatoid arthritis, or systemic lupus erythematosus, or who were receiving chronic therapies for more than 6 months per year for any diagnosis, were excluded from the analysis (35 children). Data were collected between January 2019 and June 2019. The response rate was 78%, as 4944 parents out of the 6338 who signed parental consent forms fully completed the questionnaire and their children participated in the measurements. After examination of univariate statistics to detect any anomalies in the variables’ distribution (especially outliers or missing values, aberrant values, and duplicates)—36 cases of the total—the total study sample in this analysis included 4908 secondary school children.

#### 2.2.1. Measurements

Anthropometric measurements were conducted by sixteen health professionals who were hired and trained for the purposes of this research. The children and adolescents were measured by two trained research team members, using the same equipment and protocols. Body weight was measured to the nearest 100 g using a Tanita digital scale (Tanita BWB 800ΜA). Children and adolescents were weighed without shoes, with minimal possible clothing. Height was measured to the nearest 0.1 cm, using a commercial stadiometer (Charder HM 200P Portstad). The Charder stadiometer was standardized against a Harpenden portable stadiometer. During the measurement of height, each child or adolescent stood barefoot, keeping the shoulders in a relaxed position, the arms hanging freely, and the head aligned to the Frankfort horizontal plane. Anthropometric measurements were taken three times, and the arithmetic mean of the three measurements was computed. Body weight and height were used to calculate BMI, using the Quetelet’s equation, i.e., body weight (kg)/height^2^ (m^2^). BMI was calculated, and subsequently adolescents were categorized according to the IOTF criteria [55] into the following three BMI categories: normal, overweight, and obese. Both inter-rater and intra-rater reliability, measured with intra-class correlation coefficients (ICC), yielded values greater than 0.98.

Cardiorespiratory fitness was assessed by the 20 m maximal multistage shuttle run test and maximal oxygen uptake (VO_2_max) was calculated [5]. For the calculation of VO_2_max, we used the equation of Leger et al. [5], which takes into consideration the speed at the final level (S) and the age (A) of the participant. The calculation of VO2max was carried out using the following equation:

VO_2_max = 31.025 + 3.238 × S − 3.248 × A + 0.1536 × S × A (7)

where:

A is the age and

S is the final speed (S = 8 + 0.5 × last stage completed).

#### 2.2.2. Questionnaire

A validated questionnaire [56] was used, comprising age, gender, maternal prepregnancy BMI information, and participation frequency in sports activities. These activities were defined as structured in organized athletic facilities run by the State, athletic federations, or sports and athletic clubs officially registered with the General Secretariat for Sports that reports to the Greek Ministry of Education [57]. For elementary schoolchildren, the questionnaires were completed by the parents; for high school adolescents, the questionnaires were answered by the students and the parents. The questionnaires asked pregnant girls to self-report their height and prepregnancy weight and these data were used to determine their BMI category, which can be easily collected or retrieved from medical booklets.

### 2.3. Statistical Analysis

#### 2.3.1. Descriptive and Inferential Statistics

Descriptive statistics were calculated. The results were presented as frequencies and means ±standard error, boxplots, bar plots, and scatter plots. Chi-square tests, Welch *t*-tests, Mann–Whitney-U tests, and ANOVA tests were used to test the homogeneity among boys and girls for each variable at a significance level of α = 5%. VO_2_max was measured in a ratio scale.

#### 2.3.2. Prediction-Modeling Procedure

**Figure 1:**
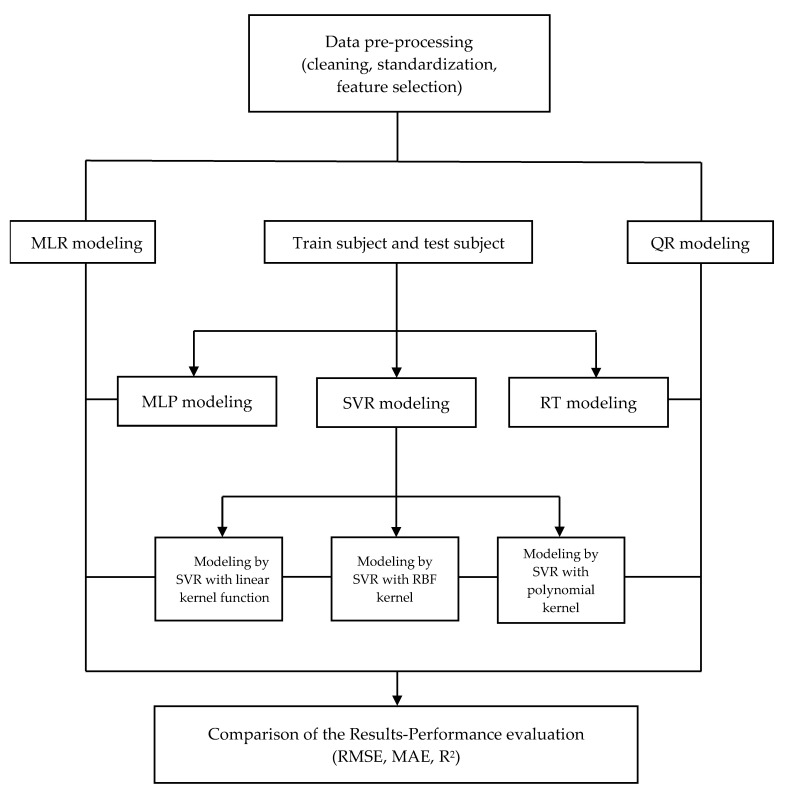
The prediction-modeling procedure of VO_2_max.

Figure 1 demonstrates the prediction-modeling procedure of VO_2_max. Data pre-processing was carried out by means of univariate statistics to detect any anomalies in the distribution of variables and data standardization, in order to eliminate the dependency of units. Train and test subsamples (90% and 10%, respectively) were used on all machine learning methods.

### 2.4. Model Selection

For MLR, a full model with main effects was fitted, and backward elimination was used to obtain a reduced model. The variables associated with the outcome variable at <0.05 level were maintained in the final models. The Akaike information criterion (AIC) was used to select the best-fit model [37].

For quantile regression, the Huber–White or sandwich estimator of variance was used in the models and the quantiles summarized were 0.10, 0.25,0.50, 0.75, and 0.90. The Akaike information criterion (AIC) was used to select the best-fit model [38].

For ridge regression, a 10-fold cross validation was used for RR in order to identify the lambda value that produced the lowest test mean squared error that led to λ values that performed a shrinkage of the coefficients [42].

For SVR, a ReliefF feature selector was used to predict and identify the relevant and irrelevant covariates of VO_2_max. The rationale was to remove the redundant and irrelevant variables and to construct a parsimonious model [33]. Table 1 indicates the ReliefF scores of the different independent variables for boys and girls, respectively. Height, weight, BMI category, age, track, and football were the more relevant features for boys; weight, BMI category, track, basketball, age, height, swimming, and maternal prepregnancy BMI were the most relevant features in girls.

Table 2 indicates the prediction models built for VO_2_max, based on the previous results. By excluding the variable with the lowest score from the full set of predictor variables iteratively, various models were constructed to predict VO_2_max.

Hyperparameter tuning was carried out by performing a grid search over specified parameter ranges for RBF, polynomial, and linear kernel functions with different penalty coefficients: cost (C) (2^–2^,2^18^), Epsilon (ε) [0.001,160], Gamma (γ) [2^−10^,29], and degree (d: 1, 2, 3, 4). A 10-fold cross-validation was used to assess the generalization error of the prediction model. Then, a comparison of the performance of these three types of kernel functions, in combination with the optimal parameters, was conducted.

For MLP, we compared the performance of different cross-validated networks, with three to eight hidden neurons, and chose the number that yielded the lowest prediction error. The rectified linear activation function, or ReLU activation function, which is perhaps the most common function, was used for the hidden layers. We performed a sensitivity analysis to investigate the effect of each variable on VO_2_max using the MLP by changing the value of one input variable while maintaining the other variables constant.

For boosting RT (RT), the start number of trees of the model was adjusted to 1 and the number of the trees was iteratively increased by 1 until a maximum number of 1000 was reached. The split was based on the sums of squares (minimum size node to split: [2,30], depth of individual trees: [5,20]. The optimal number of the trees was identified when the value of the pseudo-residuals was minimal, based on the complexity parameter. The complexity parameter was set to 0.01 and the shrinkage factor was set to 0.1. The overall importance of the independent variables, according to their roles as surrogates to the primary split, was expressed by the values of the improvements that were summed over each node, totaled, and scaled relative to the best performing variable, expressed as a relative importance on a 0–100% scale [54].

#### Performance-Evaluation Criteria among the Models

The measures of performance used in comparing the different regression models were coefficient of determination R^2^, mean absolute error (MAE), and root mean square error (RMSE). These formulae are shown below:

R^2^ = 1^-^RSS/TSS (8)

where RSS is the regression sum of squares (RSS) and TSS is the total sum of squares:

MAE = 1/n * Σ|y_i_ − ŷ_i_| (9)

where

y_i_ is the observed value for the ith observation,

according to their respective scores ŷi is the predicted value for the ith observations, and

n is the sample size:

RMSE = √Σ(y_i_ − ŷ_i_)^2^/n (10)

Statistical analyses were carried out using R language (Version 4.1.3), packages e1071, MASS, FSelector, caret, quantreg, glmnet, ridge, hydrogof, AICcmodavg.rpart, Neuralsens, gbm and rpart.plot.

## 3. Results

### 3.1. Sample Characteristics

Table 3 presents descriptive statistics by sex. Missing values did not surpass 4% in any variable measured. The majority of girls (72.2%) had not reached menarche. For the purpose of analysis, the variable of physical activity participation was dichotomized as follows: “Yes—participated in organized team or individual sports at least twice per week” and “No—did not participate in organized teams or individual sports at least twice per week” since September of the current school year [57]. The prevalence of participation in organized sports activities was significantly higher (x^2^ = 5.11, df = 1, *p* = 0.024) in boys (57.3%) than in girls (54%). In addition, boys were more likely than girls (x^2^ = 23.800, df = 2, *p* < 0.001) to be overweight (27.6% vs. 21.9%%) and obese (8.2%% vs. 5.0%).

**Figure 2:**
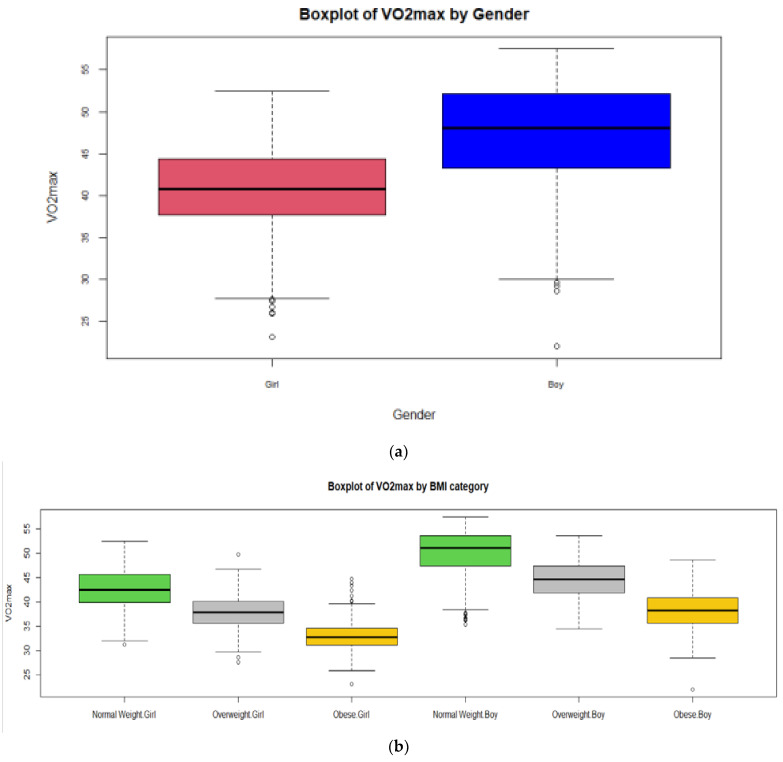
Boxplot of VO_2_max by gender (**a**) and by BMI category (**b**).

As expected, VO_2_max levels were lower for girls than for boys and these differences were highly significant (t = −43.68, df = 4906, *p* < 0.001). The distribution of VO_2_max (Figure 2) was more symmetric in girls, although both distributions presented outliers (shown at the end of the left whisker). As can be seen, normal weight boys and girls presented higher levels of VO_2_max than overweight and obese boys (F = 844.64, df = 2, *p* < 0.001) and girls (F = 980.76, df = 2, *p* < 0.001), respectively. In addition, as expected, girls in each ΒΜΙ category presented lower levels of VO_2_max than boys (F = 1416, df = 5, *p* < 0.001). Girls played volleyball more frequently (U = 410,118, *p* < 0.001) than boys and boys played basketball (U = 533,268, *p* < 0.001) and football (U = 288,862, *p* < 0.001) more frequently than girls.

**Figure 3:**
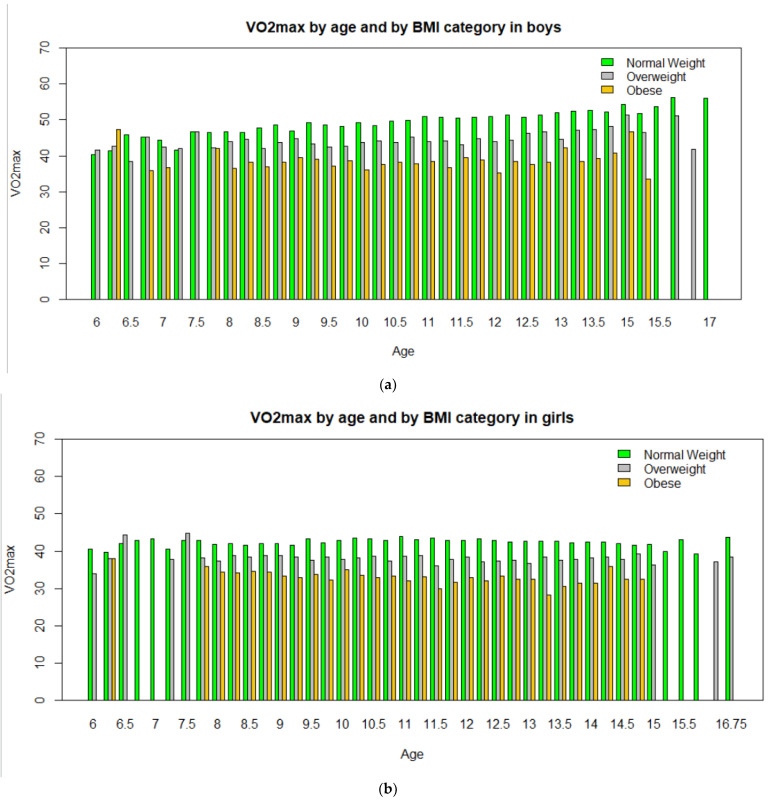
VO_2_max by age and BMI category in boys (**a**) and girls (**b**).

Figure 3 presents VO_2_max levels by age and BMI category for boys and girls, respectively. As can be seen, in both sexes, obese children and adolescents presented lower levels of VO_2_max in all ages and, according to ANOVA testing, these differences were highly significant (*p* < 0.001). Boys of normal weight showed a gradual increase in VO_2_max, from 47.0 ± 0.101 to 53.7 ± 0.083 mL.kg^−1^.min^−1^ between the ages of 8 and 17. Overweight boys showed a relatively smaller increase in VO_2_max, from 43.3 ± 0.144 to 48.2 ± 0.110 mL.kg^−1^.min^−1^ between the ages of 8 and 14. In contrast, for obese boys, VO_2_max was lower and remained unchanged (average: 38.5 ± 0.286 mL.kg^−1^.min^−1^, U = 194, *p* = 0.40) in the ages from 8 to 15. Girls with normal weight showed a significant increase in VO_2_max, from 42.0 ± 0.080 mL.kg^−1^.min^−1^ from 7 to 8 years to 43.4 ± 0.089 mL.kg^−1^.min^−1^ at 11 years (U = 480, *p* = 0.013), which was followed by a significant decrease, to 41.5 ± 0.890 mL.kg^−1^.min^−1^ at the age of 15 (U = 594, *p* = 0.002), and a new increase at the age of 16 (42.0 ± 0.980). Overweight girls showed a significant decrease (U = 499, *p* = 0.012) in VO_2_max from the ages of 7 years to 8 years (38.0 ± 0.367) and an increase in VO_2_max after the age of 15 (39 ± 0.090), but overweight girls remained in lower levels of VO_2_max than normal-weight girls across the ages examined (average: 38.0 ± 0.067 mL.kg^−1^.min^−1^), while obese girls showed a gradual decrease in VO_2_max, from 34.6 ± 0.320 to 31.6 ± 0.240 mL.kg^−1^.min^−1^, between the ages of 8 and 15 (U = 5190, *p* = 0.018).

**Figure 4:**
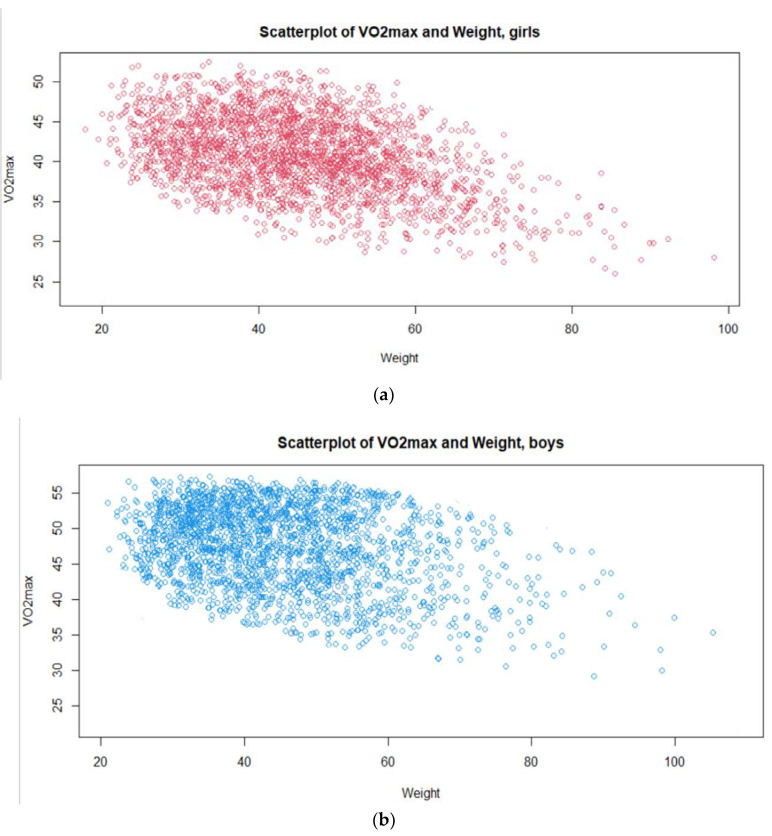

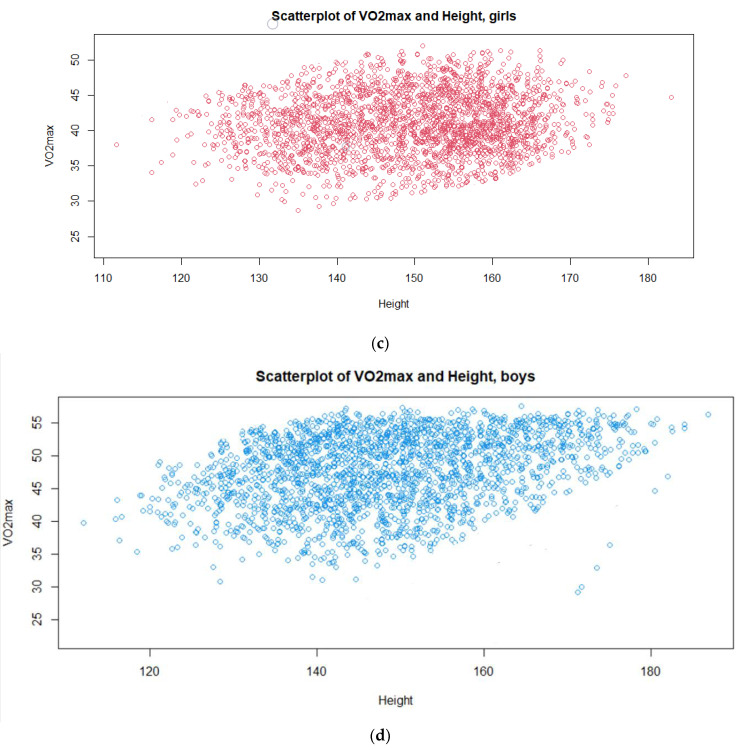
Scatterplots between VO_2_max and two continuous variables, weight (**a**) girls and (**b**) boys and height for (**c**) girls and (**d**) boys.

Figure 4 presents the scatterplots between VO_2_max and two continuous variables, weight (a) girls and (b) boys and height for (c) girls and (d) boys, indicating a negative relationship for the weight variable and a positive relationship for the height variable. In addition, the figures indicate that the assumption of a linear relationship between these variables may be supported by the data.

### 3.2. Model Results and Performance

Model 6 variable combinations for boys and model 4 variable combinations for girls (Table 3) presented the best performances in all models. Table 4 shows the R^2^, MAE, and RMSE values of VO_2_max prediction models built by combining the above-mentioned variables. As can be seen, the MLR method for boys outperformed the other methods in all performance indices, followed by SVR with linear kernel. MLP outperformed both SVR with polynomial kernel, and QR but it was outperformed by MLR, SVR-linear, and SVR-RBF, as well as by RT in terms of R^2^. SVR-RBF in girls presented better MAE and RMSE, but was outperformed by MLR in terms of R^2^, followed by SVR linear, which presented better performances than MLP, RR, and SVR with polynomial kernel. SVR-polynomial and QR presented the worst performances in all indices.

**Figure 5:**
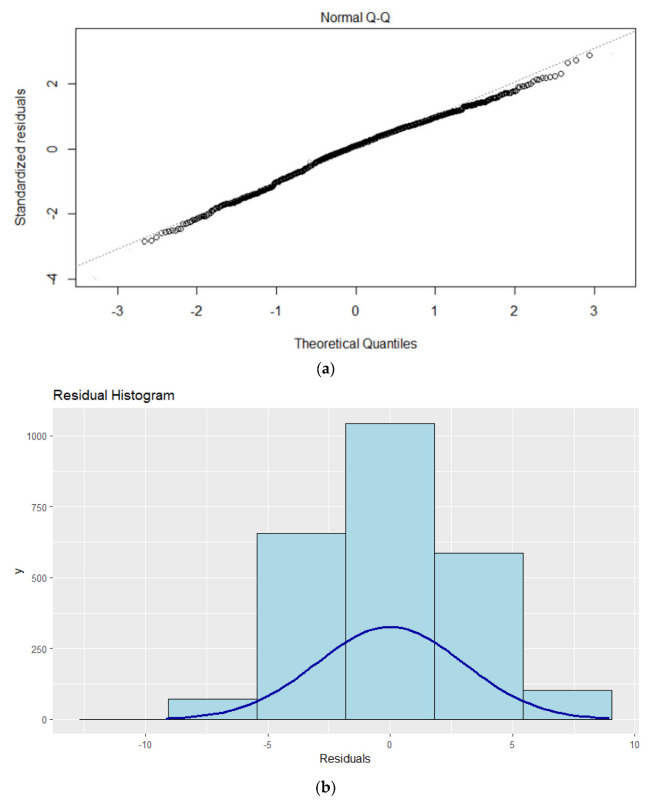

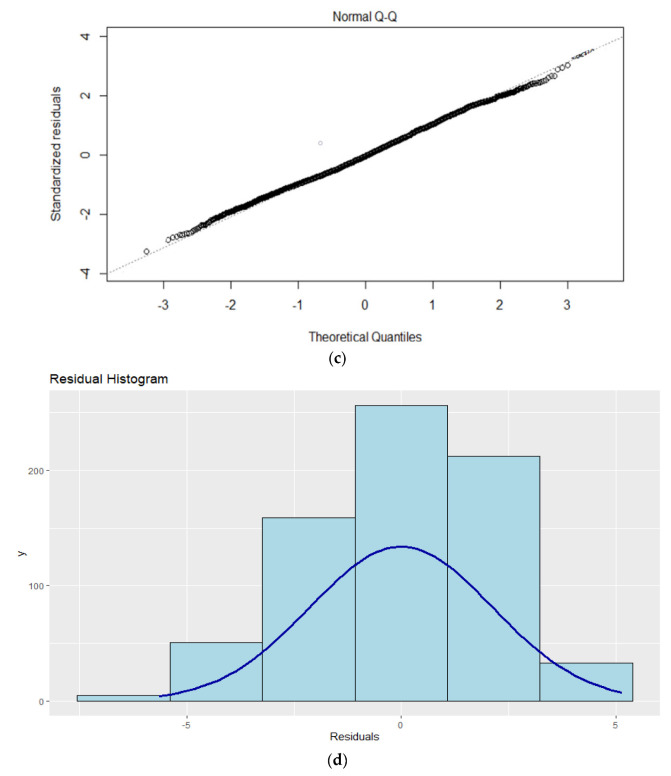
MLR residual diagnostics plots for (**a**): normal Q-Q plot, and (**b**) histogram of the residuals for boys (**c**): normal Q-Q plot, and (**d**) histogram of the residuals.

Table 5 presents the results of MLR model for boys with the smaller AIC value (AIC = 3144), and for girls ((AIC = 10,043). As can be seen from the Figure 5, the Q-Q plot and histogram indicate that residuals meets homoscedasticity and are approximately normal. As can be seen in Table 2, multicolinearity is not a serious concern (the variance inflation factor (VIF) < 10 in all cases).

#### 3.2.1. Boys

As can be seen, MLR for boys outperformed the other methods in all performance indices, followed by SVR with linear kernel. RR outperformed MLP, SVR with polynomial kernel, RT, and QR, but was outperformed by MLR, SVR-linear, and SVR-RBF in all indices. SVR-RBF MLP outperformed SVR with polynomial kernel; RT outperformed SVR-polynomial and QR and presented better R^2^ than MLP but was outperformed by MLR, SVR-linear, and SVR-RBF. The retained model for boys (with unstandardized coefficients) is as follows:

*VO2max* = −6.638 × *Obese* – 2.888 × *Overweight* + 0.858 × *Age* + 0.478 × *Football* – 0.368 × *Weight* + 0.278 × *Height* + 0.008 × *Track* + 13.599 (11)

As can be seen, being an overweight boy decreased the mean VO_2_max by 2.888 units compared with the mean VO_2_max for normal-weight children and adolescents, holding all other factors constant. Being an obese boy decreased the mean VO_2_max by 6.638, as opposed to the mean VO_2_max for normal-weight children and adolescents, holding all other factors constant. For every one-standard deviation increase in age, VO_2_max increased by 0.262 units. For every one-standard deviation increase in weight, VO_2_max decreased by 0.879 units, holding all other predictors constant. For every one-standard deviation increase in the frequency of football training per week, VO_2_max increased by 0.103 units, holding all other covariates constant. For every one-standard deviation increase in the frequency of track and field training per week, VO_2_max increased by 0.073 units, holding all other factors constant. Swimming and basketball did not remain significant in the final model, although the frequency of football and track and field training per week remained highly significant. In addition, the indices of maternal prepregnancy obesity/overweight, organized sports participation (as a dichotomous variable), volleyball training per week, and martial arts participation per week were not significant in any of the alternative models that were attempted.

#### 3.2.2. Girls

SVR-RBF in girls presented better MAE and RMSE, but was outperformed by MLR in terms of R^2^, followed by SVR-linear that presented better performances than MLP, RR, RT, SVR with polynomial kernel, and QR.

For girls, the MLR model (with unstandardized coefficients) with the lowest value of Akaike information criterion (AIC = 10,144) was the following:

*VO_2_max* = −2.646 × *Obese* − 1.219 × *Overweight* + 0.566 × *Basket* + 0.435 × *Track*− 0.337 × *Weight* − 0.303 × *Maternal* (12)

*Prepregnancy obesity/overweight* + 0.234 × *Height* + 0.224 × *Age* + 0.131 × *Swimming* + 18.88

Based on this model, being an overweight girl decreased the mean VO_2_max by 1.219, compared to the mean VO_2_max for normal-weight girls, holding the other predictors constant. Being an obese girl decreased by 2.646 units the mean VO_2_max, compared with normal-weight girls. The mean VO_2_max for girls with maternal prepregnancy obesity/overweight was reduced by 0.303 units, as opposed to the mean VO_2_max for normal-weight girls, holding the other predictors constant. For every one-standard deviation increase in age, VO_2_max increased by 0.224 units. For every one-standard deviation increase in weight, VO_2_max decreased by 0.337 units. For every one-standard deviation increase in the frequency of basketball training per week, VO_2_max increased by 0.566 units, holding all other predictors constant. For every one-standard deviation increase in the frequency of track and field training per week, VO_2_max increased by 0.435 units. For every one-standard deviation increase in the frequency of swimming training per week, VO_2_max increased by 0.131 units, holding all other predictors constant. The indices of the menarche group, organized sports participation (as a dichotomous variable), and football training per week were not significant in any of the alternative models attempted, while volleyball training did not remain significant in the final model.

In addition, Table 5 presents linear SVR featuring weight results by gender. As can be seen, weight and obesity presented higher values in both sexes.

Moreover, the best SVR models (Table 4) comprised the same predictors that were included in the final MLR models

**Figure 6:**
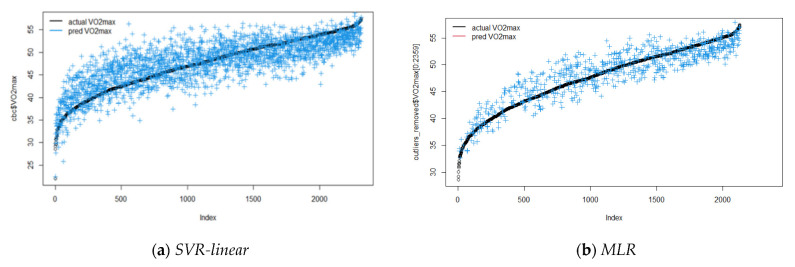
Scatterplots for actual and predicted values using SVR-linear (**a**) and MLR (**b**) for boys (tuned model).

**Figure 7:**
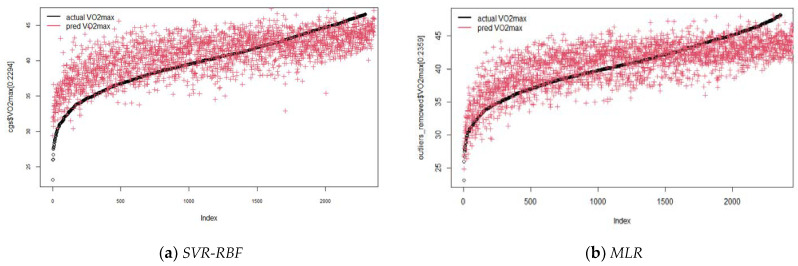
Scatterplots for actual and predicted values using SVR-RBF and MLR for girls (tuned model): (**a**) SVR-RBF; (**b**) MLR.

Figure 6 and Figure 7 present the values of VO_2_max predicted by the tuned models for boys (C = 4, epsilon = 0.1) and for girls (C = 8, epsilon = 0.1, γ = 0.08), compared with MLR. For RR, the coefficients are presented in Table 6. Obesity and being overweight presented higher significant coefficients in both sexes.

Figures S1 and S2 Supplementary Materials indicate mean square error (MSE) by lambda (λ) value and ridge trace plot for boys and girls, respectively. We can see that decreasing the values of λ led to the shrinkage of the regression coefficients; some of these even became zero. The best value of λ for boys that minimizes the MSE was 0.326, while the best value of lambda for girls was 0.294. It should be mentioned that MSE minimization was obtained by the same covariates included in final MLR and SVR models. Table 6 shows the coefficients of RR.

**Figure 8:**
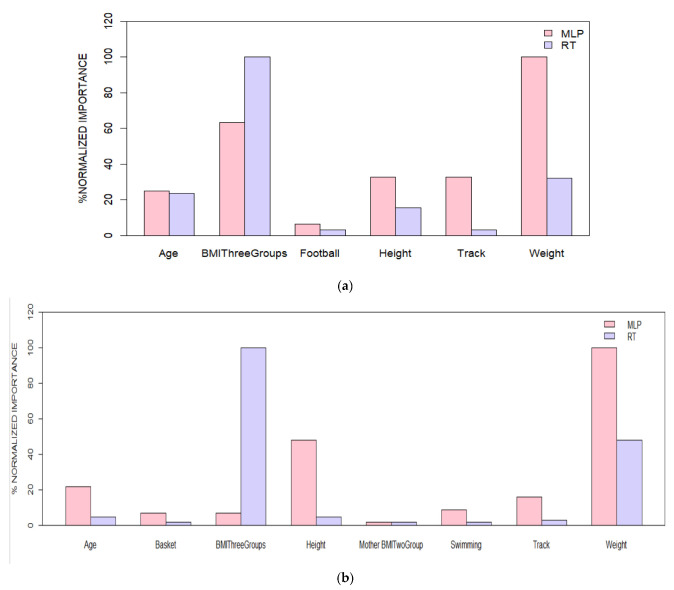
Variable importance (normalized). MLP and RT models for (**a**) boys and (**b**) girls, with the percentages of normalized importance for MLP and RT for both boys and girls.

Figure 8 presents the relative importance of variables, comparing MLP and RT for boys (a) and girls (b). As can be seen, according to all surrogate splits in RT and the sensitivity analysis in MLP, the most important variables were the BMI category and weight. Variable combinations of model 6 for boys and model 4 for girls presented the best performances. The weight and the BMI category remained the most importable variables in all models.

Figures S3 and S4 Supplementary Materials show the effect of each of the explanatory variables on VO_2_max and the coefficients, by quantile, of the QR model and the MLR for boys and girls, respectively, with necessary explanations. The quantiles of the response variables are on the horizontal axis and the coefficients’ magnitudes are on the vertical axis. The red line represents the ordinary least squares regression coefficient, and the red dotted lines represent the 95% confidence interval of the ordinary least squares regression coefficient. The dotted black line represents the quantile regression coefficients. The gray boundaries represent the 95% confidence intervals of the respective quantile regression coefficients. The regression coefficients of the predictors were in the majority of the quantiles within the 95% confidence intervals of the traditional regression. except for some coefficients in the low (10th) and high quantiles (90th), possibly due to the heterogeneity of the data as such (e.g., weight and height). The two models attempted the minimum Akaike information criterion (boys AIC: 8434, girls AIC: 11,043) values by using in each model (or adjusting for) the same variables that appeared as significant in the MLR and SVR models. In addition, at the 75th percentile that presented the minimum RMSE in girls, compared to other percentiles, the significant covariates were identical with those of the MLE, SVR and RR models (Table 4). Τhe same holds true for boys at the 50th percentile that presented the minimum RMSE (Table 4).

Nevertheless, the relative importance (absolute values) of the corresponding weights was almost similar to that of the MLR analysis, which increases the evidence of the relationships emerging for the relationship between VO_2_max and these covariates. In addition, it must be mentioned that in all models, obesity/overweight was a highly significant risk factor, having a negative impact on the levels of VO_2_max.

## 4. Discussion

There are many studies that have shown the association of increased BMI with impaired physical fitness. However, there are limited objective data assessing the level of cardiorespiratory fitness in children and adolescents and evaluating the impact of sex, being overweight, and obesity and, even more, the impact of parental obesity on the level of children’s cardiorespiratory fitness. In this study, an objective measurement was performed, using the 20 m multistage shuttle run test and calculating VO_2_max.

After applying different regression models of different complexity, and by using performance indicators such as RMSE, MAE, and R^2^, we concluded that the classical MLR model performs better than more complex models, such as QR, RR, and RT, and competed with SVR-RBF, which outperforms it in all indicators in the boys’ model and in R^2^ in the girls’ model and similar results were found in the literature [58]. These findings are consistent with other findings in the literature, as we have large-scale data with only a few outliers, with normality of residual distributions, and with acceptable levels of multicolinearity [59,60].

Despite its limitations, MLR led to a more accurate and precise understanding of the association of each individual factor with the outcomes than some ML methods, such as MLP or even SVR-RBF, which presents, in general, a higher predictive power but is sufficiently complex and not straightforwardly interpretable. It must be noted that SVR-linear performed better than SVR-RBF in the sample of boys, competed with ΜLR in boys, and competed with SVR-RBF and MLR in girls. Similar results are found in the literature in cases of large samples/approximately normal data [61]. Although in SVR-linear feature weights are directly interpretable, in general only SVR-RBF was used to predict VO_2_max, probably because of its well- known predictive accuracy. Nevertheless, the formulas for RBF cannot provide a weight for the features.

The polynomial kernel, which performed poorly in this study, heuristic methods may identify the features that have the highest weights, but without explicitly constructing all weights. In addition, models that are more interpretable, such as regression-based trees, performed poorly and these results were consistent with those of other studies in the field [34]. A lack of interpretability in predictive models can undermine trust in these models, especially in the field of health, in which clarity of decisions is obviously necessary. In addition, an important finding in our study was the stability of covariates’ statistical importance across the models, which increased the evidence of the associations between VO_2_max and these covariates.

The application of statistical models revealed that age, type of physical activity undertaken on a regular basis, and degree of participation, as assessed by the frequency of training, influenced VO_2_max in both sexes. The objective determination of this association supported the notion that engaging children in specific sport activities, such as football, track and field, swimming, and basketball, increases their levels of cardiorespiratory fitness. These findings broadly support the results of other studies [22,23,24,25]. Furthermore, higher training frequency had an additive effect. The effect of these different sports activities on VO_2_max was influenced by different preferences between the sexes in regard to their choices of physical activities [62]. Frequency of martial arts training per week and frequency of volleyball training per week did not present as significant in either sex, adjusting for the other sports activities and the other covariates. These results are consistent with those of other studies [21,25]. Frequency of basketball training per week and frequency of swimming training per week remained as important variables in the final models only for girls. Moreover, frequency of track and field training per week remained of significant importance for both sexes in the final models, but its positive effect seemed to be stronger for girls. Previous studies showed that although there is compelling evidence that prenatal hormones play a role in the differences between the sexes, in terms of sports interest and motivation, there is thin evidence for the role of socialization [63]. Absolute VO_2_max increased with age in all groups, with the increase being more prominent in boys than in girls, a finding that was previously reported in other studies, independently of volumes of systematic endurance training [16].

Height and weight were significant predictors of VO_2_max in both sexes and these findings were consistent with those of other studies [1,28,29,30,31,32,34]. Increased BMI seemed to be a crucially important risk factor for decreased levels of cardiorespiratory fitness in all statistical models that were attempted in this study, and this finding is also supported by other studies. [64,65,66,67]. The finding that increased BMI was associated with decreased cardiorespiratory fitness indicated that the basis for cardiometabolic risk in adulthood is already set in childhood. It will be of great interest to conduct a prospective study that measures VO_2_max in children and adolescents following nutritional intervention to lower their BMI, without an increase in the time spent for physical activity.

Available data suggest that prevention of obesity in women of reproductive age is widely recognized to be important not only for their own health, but also for the health of their children [68]. Of great interest in this study is the finding that maternal prepregnancy BMI influences the level of physical fitness of girls, clearly indicating a gender difference in vulnerability and implicating a possible genetic factor that simultaneously affects or connects the degree of adiposity and the level of physical activity. A genetic trait may influence both physical activity and sedentary behaviors; furthermore, such a trait can determine family habits regarding involvement in physical activity or having a sedentary lifestyle. It is known that parental obesity is a risk factor for developing obesity in offspring [68]. The current data highlight another risk associated with maternal obesity—that of poorer cardiorespiratory fitness of their children.

Plaza-Florido et al. have recently investigated molecular mechanisms that influence cardiorespiratory fitness in children who are overweight or obese, and they reported that differentially expressed genes between fit and unfit children enriched dopaminergic and GABAergic synapse pathways and that most of the genes were upregulated in these pathways [69]. Furthermore, several studies have concurred with the present finding [13,70,71].

An optimistic result is that the mean estimated VO_2_max is higher than the new international criterion-referenced standards of 42 mL/kg/min (boys) and 35 mL/kg/min (girls) for healthy CRF [72], while the Eurofit data revealed a latitudinal gradient, where children and adolescents from north-central Europe typically have better CRF than their peers from southern Europe [73].

This study presents several advantages. By comparing traditional methods, such as MLR and QR, with “white box” regression-based methods, such as RR and RT, and “black box” regression-based methods, such as MLP and kernels-based SVR, stratified by gender, we interpreted interesting results. First, important results of this study include the superiority of MLR in all performance indicators in the case of boys; the outperforming by MLR of more “sophisticated” methods, such as “white box“ regression-based ML methods in all cases; and “black box machine learning methods” in terms of R^2^ in all cases. This fact, combined with the undoubtedly more accurate and precise understanding of the association between each individual factor and the outcomes arising from MLR, indicate that in the absence of data-based evidence, researchers must not exclude this method as a candidate in their analysis plan. Second, the same is true for SVR with linear kernel, which presents in this study with better performance than SVR-RBF in the case of boys. This fact, in combination with the possibility of direct interpretation of SVR-linear features, indicate that this method, which is less explored in recent literature, must not be excluded from an SVR-analysis plan, especially in the case of large samples of approximately normal data.

The third advantage is the stability of covariates’ statistical importance across all models, which increases the evidence of the emerging associations between VO_2_max and these physiological and sports activities characteristics. Fourth, the differentiation of the covariates influencing VO_2_max in boys and girls, such as the maternal prepregnancy BMI category, as revealed by our stratified-by-gender analysis, constitutes a finding that has been less explored in the current literature, and which needs further exploration.

Limitations of the present study include the following: (1) the self-reporting of information regarding sports participation, which is a source of bias; (2) the fact that we have taken into account only the organized sports activities practiced in this sample; and (3) the self-reporting of maternal prepregnancy BMI. Nevertheless, previous studies indicated that there was a strong agreement between self-reported weight and the weight measured in the first trimester of pregnancy [74].

Strong points of this study are the nationwide participation and the large number of the cohort, which increased the external validity of the study.

Prevention is the cornerstone of addressing the epidemic of childhood obesity. The evidence for impaired cardiorespiratory fitness being associated with being overweight or obese in childhood may be used as an easy and low-cost screening measure that will support the need for lifestyle interventions and possibly reveal the effect of such interventions prior to the achievement of a normal BMI for age and sex. Addressing cardiorespiratory fitness in children and adolescents could reduce future adiposity and become an important factor in improving health. A greater public awareness of the need for action, at the levels of both individuals and society, to address the unmet needs in the prevention of obesity during girls’ preconception periods is evident. In addition, parents, especially those with children of early school ages, provide a target group for interventions that are suitable for education regarding the importance of preventing being overweight or obese and incentives to promote sports activities for their children, either by acting as behavior models or by supporting their child’s active participation in sport activities.

Collaboration of physical educators and scientists of exercise physiology with pediatricians and pediatric endocrinologists treating children with increased adiposity is of the utmost importance for an optimal outcome. Furthermore, their input in planning large scale prevention strategies, as members of multidisciplinary teams, is necessary.

Predictors that are not considered modifiable, such as age and gender, can be used to guide targeted interventions and policies.

## Figures and Tables

**Table 1:** Relief score of the independent variables and VO_2_max.

Boys		Girls	
Variables	ReliefF Score	Variables	ReliefF Score
Height	0.00293	Weight	0.01688
Weight	0.00259	BMI category	0.01580
BMI category	0.00189	Track	0.01502
Age	0.00099	Basketball	0.00882
Track	0.00070	Age	0.00800
Football	0.00045	Height	0.00785
Basketball	0.00004	Swimming	0.00391
Swimming	0.00003	Maternal prepregnancy BMI category	0.00389
Volley	−0.00495	Menarche	−0.00001
Martial arts	−0.00560	Physical activity participation	−0.00001
Physical activity participation	−0.00804	Volleyball	−0.00045

**Table 2:** Prediction models built for VO_2_max.

Boys		Girls	
Models	Variables	Models	Variables
Model 1	Height, Weight, BMI category, Age, Track, Football, Basketball, Swimming, Volley, Martial arts, Physical activity participation	Model 1	Weight, BMI category, Track, Basketball, Age, Height, Swimming, Maternal prepregnancy BMI category. Menarche, Physical activity participation, Volleyball
Model 2	Height, Weight, BMI category, Age, Track, Football, Basketball, Swimming, Volleyball, Martial arts	Model 2	Weight, BMI category, Track, Basketball, Age, Height, Swimming, Maternal prepregnancy BMI category. Menarche, Physical activity participation
Model 3	Height, Weight, BMI category, Age, Track, Football, Basketball, Swimming, Volleyball	Model 3	Weight, BMI category, Track, Basketball, Age, Height, Swimming, Maternal prepregnancy BMI category. Menarche
Model 4	Height, Weight, BMI category, Age, Track, Football, Basketball, Swimming	Model 4	Weight, BMI category, Track, Basketball, Age, Height, Swimming, Maternal prepregnancy BMI category
Model 5	Height, Weight, BMI category, Age, Track, Football, Basketball	Model 5	Weight, BMI category, Track, Basketball, Age, Height, Swimming
Model 6	Height, Weight, BMI category, Age, Track, Football	Model 6	Weight, BMI category, Track, Basketball, Age, Height
Model 7	Height, Weight, BMI category, Age, Track	Model 7	Weight, BMI category, Track, Basketball, Age
Model 8	Height, Weight, BMI category, Age	Model 8	Weight, BMI category, Track, Basketball
Model 9	Height, Weight, BMI category	Model 9	Weight, BMI category, Track
Model 10	Height, Weight	Model 10	Weight, BMI category
Model 11	Height	Model 11	Weight

**Table 3:** Sample descriptive statistics.

Variables (Codes)	Boys N = 2314 (47.15)	Girls N = 2594 (52.85)
	N (%)	N (%)
BMI		
Underweight/Normal Weight (0)	1486 (64.2)	1824 (69.7)
Overweight (1)	639 (27.6)	574 (21.9)
Obese (2)	209 (8.2)	130 (5.0)
Missing	-	89 (3.4)
Maternal Prepregnancy BMI group		
Normal Weight (0)	1389 (62,4)	1515 (58.4)
Overweight/obese (1)	838 (37.6)	976 (39.2)
Μissing	-	103 (3.97)
Menarche group		
No (0)	-	1874(72.2)
Yes(1)	-	520(27.8)
Organized sports participation		
No (0)	989 (42.7)	1192 (46.0)
Yes(1)	1325 (57.3)	1402 (54.0)
Variables	Mean ± standard error	Mean ± standard error
VO_2_max	47.391 ± 0.121	40.843 ± 0.092
Age	11.71 ± 0.038	10.78 ± 0.039
Height	148.732 ± 0.274	149.080 ± 0.236
Weight	45.147 ± 0.276	45.113 ± 0.242
Football training per week (times)	1.76 ± 0.035	0.67 ± 0.018
Basketball training per week (times)	1.120 ± 0.028	0.760 ± 0.020
Track and field training per week (times)	0.309 ± 0.019	0.380 ± 0.020
Swimming training per week (times)	0.740 ± 0.022	0.730 ± 0.021
Martial arts training per week	0.420 ± 0.030	-
Volleyball training per week (times)	0.680 ± 0.190	1.110 ± 0.027

**Table 4:** Performance evaluation by method.

Boys				Girls		
Method	R^2^	MAE (ml.kg^−1^.min^−1^)	RMSE (ml.kg^−1^.min^−1^)	R^2^	MAE (ml.kg^−1^.min^−1^)	RMSE (ml.kg^−1^.min^−1^)
MLR	0.831	1.747	2.133	0.590	2.467	3.010
SVR-RBF	0.745	2.720	3.400	0.580	2.400	2.840
SVR-linear	0.760	2.681	3.330	0.503	2.398	2.993
RR	0.674	2.556	3.099	0.469	2.690	3.337
MLP	0.545	2.840	3.100	0.495	2.565	3.084
RT	0.600	2.999	3.170	0.324	3.004	3.540
SVR-polynomial	0.535	4.520	4.000	0.435	4.690	5.131
QR	0.542	4.290	4.321	0.430	3.600	4.170

**Table 5:** MLR results and SVR-linear feature weights by sex.

	BOYS							GIRLS						
	Coefficient (95%CI)	Standard Error	Coefficient ^1^	*t* Stat	*p* Value	*VIF*	*Feature Weights W* ^1^	Coefficient (95%CI)	Standard Error	Coefficient ^1^	*t* Stat	*p* Value	*VIF*	*Feature Weights W ^1^*
Predictors														
Intercept	13.509 (9.921;17.097)	1.829		7.368	<0.001		0.279	18.882 (15.997;21.798)						0.181
Overweight versus normal weight	−2.888 (−3.397;−2.379)	0.270	−2.888	−10.355	<0.001	1.015	−0.574	−1.219 (−1.640;−0.756)	0.231	−1.219	−5.275	<0.001	3.460	−0.316
Obese versus normal weight	−6.638 (−7.547;−5.723)	0.480	−6.638	−13.339	<0.001	1.041	−0.681	−2.646 (−3.465;−1.827)	0.425	−2.646	−6.228	<0.001	1.077	−0.553
Age	0.858 (0.650;1.068)	0.111	0.262	8.159	<0.001	1.324	0.292	0.223 (0.100;0.348)	0.065	0.095	3.149	0.002	1.054	0.115
Height	0.278 (0.241;0.313)	0.019	0.634	14.666	<0.001	3.137	0.583	0.235 (0.206;0.263)	0.014	0.602	15.951	<0.001	6.927	0.553
Weight	−0.368 (−0.394;−0.341)	0.015	−0.879	−25.588	<0.001	5.273	−0.927	−0.339 (−0.370;−0.307)	0.016	−0.893	−20.350	<0.001	9.340	−0.852
Football training/week	0.478 (0.309;0.446)	0.092	0.103	5.819	<0.001	1.273	0.107	NS		NS				-
Tracktraining/week	0.008 (0.0039;−0.012)	0.002	0.073	3.829	<0.001	1.029	0.020	0.436 (0.311;0.559)	0.065	0.099	6.487	<0.001	3.736	0.090
Swimming training/week	NS ^2^	-	NS	-		1.087		0.121 (0.015;0.243)	0.064	0.028	2.057	0.049	1.062	0.013
Basketball training/week	NS	-	NS			1.015		0.529	0.193	0.039	2.738	0.004	3.460	0.020
Maternal prepregnancy BMI category	NS		NS					−0.303	0.141	−0.303	−2.138	0.0326	1.077	−0.020

^1 ^Standardized for continuous variables. ^2^ Non significant.

**Table 6:** Ridge Regression Results.

	BOYS					GIRLS				
	Estimate	Scaled Estimate	Standard Error (Scaled)	*t* Stat (Scaled)	*p* Value	Estimate	Scaled Estimate	Standard Error (Scaled)	*t* Stat (Scaled)	*p* Value
Predictors										
Intercept	12.555					19.702				
Overweight versus normal weight	−3.189	−58.850	3.553	16.564	<0.001	−1.237	−29.3761	5.143	5.712	0.049
Obese versus normal weight	−6.548	−66.700	4.238	15.740	<0.001	−2.233	−49.856	6.080	8.200	<0.001
Age	0.919	50.700	5.620	9.022	<0.001	0.212	21.477	6.176	3.477	<0.001
Height	0.264	10.777	7.15	15.069	<0.001	0.2106	129.600	8.005	16.190	<0.001
Weight	−0.363	−10.521	5.744	26.745	<0.001	−0.311	−195.356	9.125	21.409	<0.001
Football training/week	0.478	17.610	3.138	5.613	<0.001	NS		NS		
Track training/week	0.007	12.141	3.242	3.745	<0.001	0.435	23.497	3.392	6.927	<0.001
Swimming training/week	NS ^1^	-	NS	-		0.117	6.473	3.426	1.889	0.050
Basketball training/week	NS	-	NS		1.015	0.519	9.110	3.366	2.707	0.007
Maternal prepregna-ncy BMI category	NS		NS			−0.308	−7.738	3.405	2.272	0.023

^1^ not significant.

## Data Availability

The data presented in this study are available on request from the from the authors team.

## Supplementary Materials

The following supporting information can be downloaded at: https://www.mdpi.com/article/10.3390/children9121935/s1, Figure S1: Plot of test MSE by lambda value and Ridge trace plot (boys), Figure S2: Plot of test MSE by lambda value and Ridge trace plot (girls), Figure S3: Quantile Regression versus Ordinary Least Squares (boys), Figure S4: Quantile Regression versus Ordinary Least Squares (girls), Table S1: Regions and Prefectures included in the sample
