# Supplementary material for: Predicting VO2max in Children and Adolescents Aged between 6 and 17 Using Physiological Characteristics and Participation in Sport Activities: A Cross-Sectional Study Comparing Different Regression Models Stratified by Gender
Source: Children (Basel). 2022 Dec 9;9(12):1935. doi: 10.3390/children9121935 (PMC9776983; doi:10.3390/children9121935)

Appendix A

Table A1. Regions and Prefectures included in the sample

Regions	Prefectures	% of sample
Eastern Macedonia Thrace	Drama	1.10
	Evros	1.10
	Kavala	1.10
	Xanthi	1.10
	Rodopi	1.10
Central Macedonia	Thessaloniki(2 regional units)	10.40
	Imathia	1.10
	Kilkis	1.10
	Pella	1.10
	Pieria	1.10
	Serres	2.20
	Halkidiki	1.10
Western Macedonia	Kastoria	1.10
	Kozani	2.20
	Florina	1.10
Epirus	Arta	1.10
	Thesprotia	1.10
	Ioannina	2.20
	Preveza	1.10
Thessalia	Magnesia	2.00
	Larissa	2.70
	Trikala	1.50
Ionian Islands	Zakynthos	1.00
	Corfu	1.10
	Kefallinia	1.00
Western Greece	Aetoloakarnania	2.50
	Ahaia	3.20
	Viotia	1.80
	Evia	2.00
	Fthiotida	1.70
Attiki	Athens (6 regional units)	20.80
	Piraeus	6.30
Peloponnese	Argolida	1.10
	Arkadia	1.10
	Corinthos	1.10
	Laconia	1.10
	Messenia	2.20
North Aegean	Lesvos	1.10
	Samos	1.10
	Chios	1.10
South Aegean	Cyclades (Samos, Santorini)	2.20
	Dodecanese (Rhodes, Kastelorizo)	2.20
Crete	Heraklion	2.60

	Lasithi	1.00
	Rethymno	1.00

Figure S1. Plot of test MSE by lambda valueand Ridge trace plot (boys)

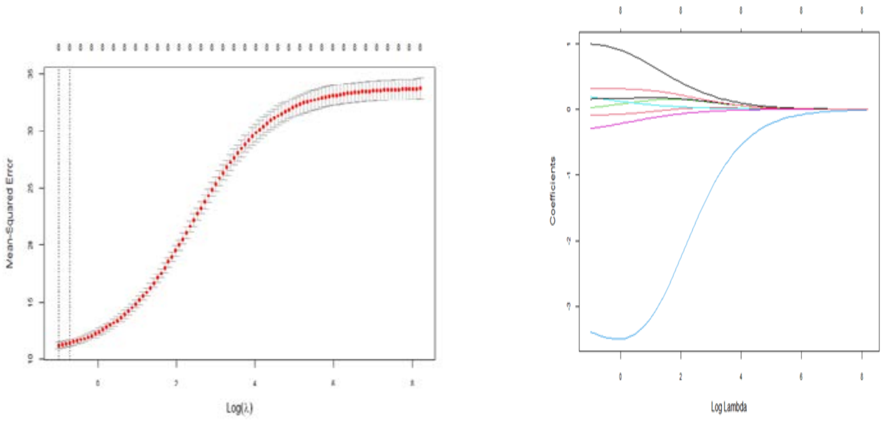

Figure S2. Plot of test MSE by lambda valueand Ridge trace plot (girls)

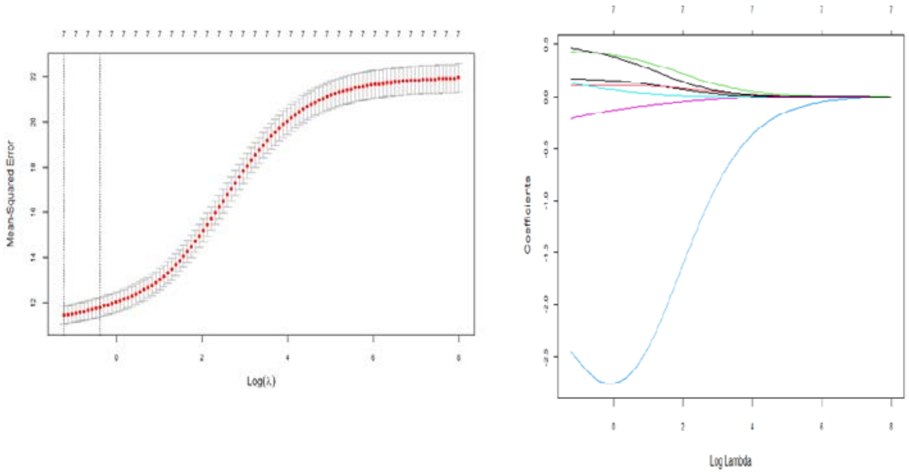

Figure S3 Quantile Regression versus Ordinary Least Squares (boys)

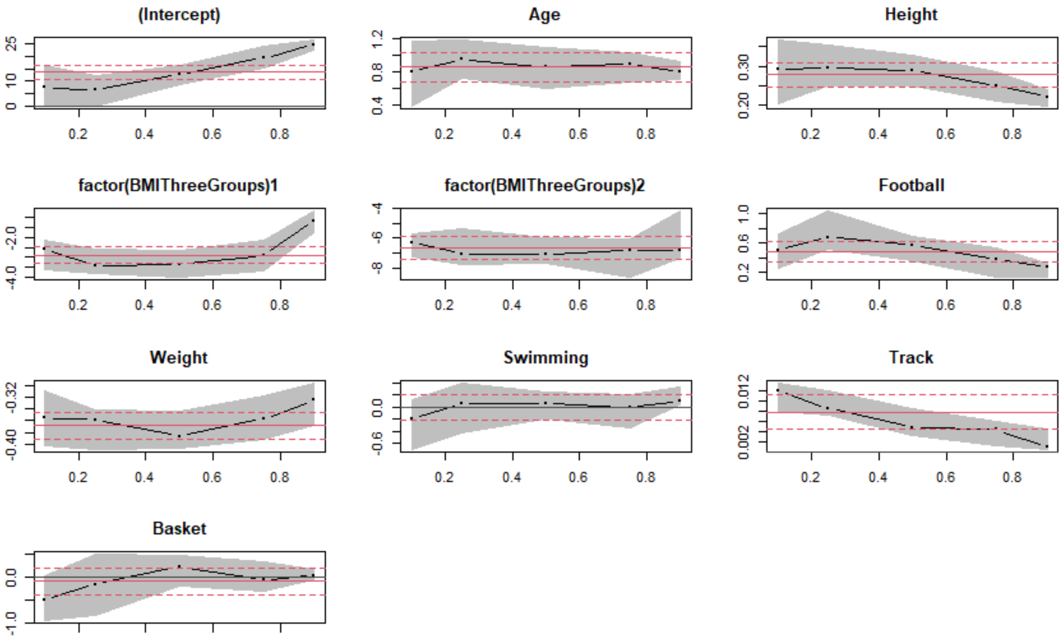

Figure S4 Quantile Regression versus Ordinary Least Squares (girls)

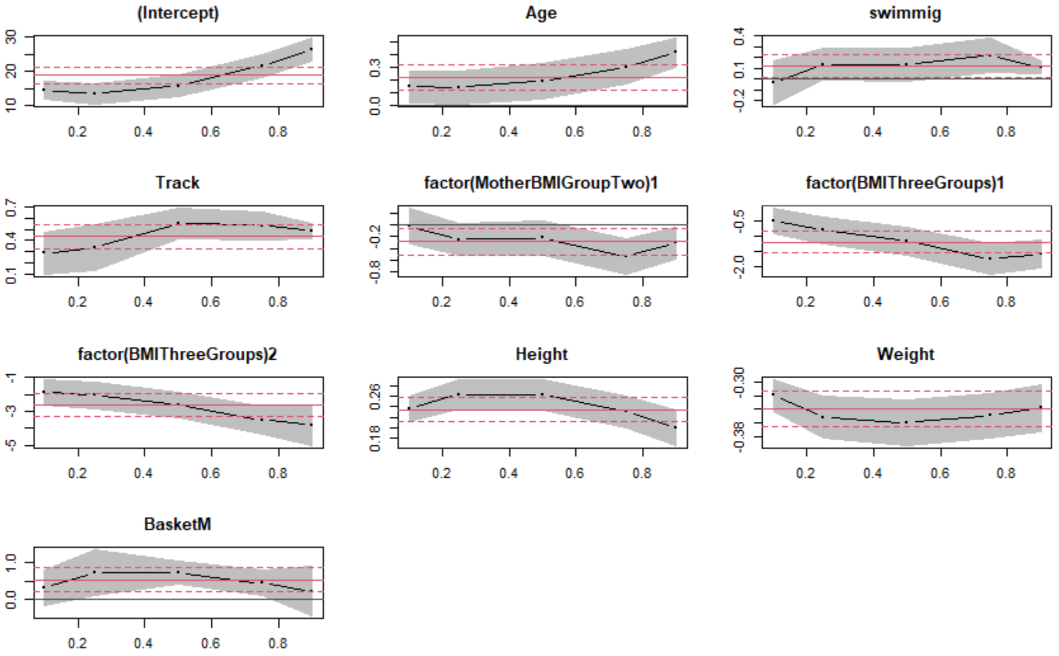

Supplement: Supplementary file 1 [file children-09-01935-s001.zip › children-2003744-supplementary.pdf]
